# Lectures on Materia Medica and Therapeutics

**Published:** 1857-01

**Authors:** 


ART. VI.—*Lectures on Materia Medica and Therapeutics.* By JOHN B. BECK, M.D., Professor of Materia Medica and Therapeutics. Prepared for the Press by C. R. GILMAN, M.D., Professor of Obstetrics, &c., in the College of Physicians and Surgeons, New York. New York, S. S. & W. Wood. 8vo. pp. 559. 1856.

THIS is the second edition of the posthumous work of the late lamented Dr. John B. Beck, one of three brothers who rendered signal service to the cause of medical science in the New World, as practitioners, writers, and teachers. It would be difficult to say which of them possessed the greatest talent, the most industry, or the greatest devotion to his profession. They were all men of the most excellent moral character, high-toned gentlemen, superior scholars, ready teachers, and accomplished writers. Dr. John B. Beck occupied for nearly a quarter of a century the chair of Materia Medica and Therapeutics in the College of Physicians and Surgeons of New York, where he died, in the winter of 1851, in the fiftysixth year of his age, from protracted disease of the bowels. He became an author almost immediately after he entered the profession, his Inaugural Dissertation on Infanticide having been published in his brother's treatise on Medical Jurisprudence, where it afterwards held a conspicuous place. We do not know any other article upon the subject so replete with scientific and valuable matter; and there is none, we are sure, which has exercised such an authoritative influence in our courts of law. Subsequently, Dr. John B. Beck published a small volume of Medical Tracts, and, at a still later period, a short treatise on Infantile Therapeutics, one of the most valuable contributions of the kind of the present day. He also edited several editions, with important additions, of Murray's Materia Medica, and a few years before he died he published a concise History of Medicine during our colonial existence.

The present work, consisting of the lectures delivered by Dr. Beck in the New York College of Physicians and Surgeons, was first issued in 1851, under the supervision of his friend, Dr. Gilman, the distinguished Professor of Obstetrics in that Institution. The fact that a new edition has been called for in so short a time shows the estimation in which the work is held by the profession. As a text-book for the student during his attendance upon lectures, we believe that it has no superior in the English language. Written in a clear, terse, and concise style, it is replete with practical instruction and sound doctrine, from the study of which no one can rise without benefit. The editor has introduced new articles on anæsthetics and cod-liver oil, and has brought up the whole work to the existing state of the science. We cordially commend the work to the attention of the profession, especially to the student and junior practitioner.

